# Incidence of Death From Unintentional Injury Among Patients With Cancer in the United States

**DOI:** 10.1001/jamanetworkopen.2019.21647

**Published:** 2020-02-21

**Authors:** Kunyu Yang, Yongqiang Zheng, Jiangtong Peng, Jiayuan Chen, Huayi Feng, Kaixu Yu, Ying Chen, Wenjing Luo, Pengcheng Yang, Yun Yang, Bian Wu

**Affiliations:** 1 Cancer Center, Union Hospital, Tongji Medical College, Huazhong University of Science and Technology, Wuhan, China; 2 Department of Cardiology, Union Hospital, Tongji Medical College, Huazhong University of Science and Technology, Wuhan, China

## Abstract

**Importance:**

Previous studies have suggested that patients with cancer may be at an increased risk of death from unintentional injury, but to our knowledge, no large studies have examined the rates of death from unintentional injury among patients with cancer.

**Objective:**

To characterize the incidence of death from unintentional injury among patients with cancer in the United States.

**Design, Setting, and Participants:**

This retrospective cohort study included patients diagnosed with a first primary cancer between January 1, 1973, and December 31, 2015, identified from the Surveillance, Epidemiology, and End Results (SEER) program data. Comparisons with the general US population were based on mortality data collected by the National Center for Health Statistics. Analyses were performed from February 1, 2019, to August 15, 2019.

**Main Outcomes and Measures:**

Rates and standardized mortality ratios (SMRs) of death from unintentional injury among patients with cancer.

**Results:**

A total of 8 271 020 patients with cancer were included in this study (50.2% female; mean [SD] age, 63.0 [15.7] years). Among them, 40 599 deaths from unintentional injury were identified. The rates of death from unintentional injury were 81.90 per 100 000 person-years among patients with cancer and 51.21 per 100 000 person-years in the corresponding US general population. The SMR of death from unintentional injury was 1.60 (95% CI, 1.58-1.61). Higher rates of death from unintentional injury were associated with increasing age at diagnosis (≥80 years; rate ratio [RR], 2.91; 95% CI, 2.84-2.98; *P* < .001), male sex (RR, 1.69; 95% CI, 1.66-1.73; *P* < .001), American Indian or Alaskan Native population (RR, 1.48; 95% CI, 1.30-1.68; *P* < .001), and being unmarried (RR, 1.23; 95% CI, 1.18-1.28; *P* < .001). Rates of death from unintentional injury were the highest in patients with cancers of the liver (200.37 per 100 000 person-years), brain (175.04 per 100 000 person-years), larynx (148.78 per 100 000 person-years), and esophagus (144.98 per 100 000 person-years). The SMRs were the highest in the first month after cancer diagnosis.

**Conclusions and Relevance:**

This study found that the incidence of death from unintentional injury among patients with cancer was significantly higher than that in the general population in the United States. The rates of death from unintentional injury varied by age, sex, race/ethnicity, marital status, cancer site, disease stage, and time since diagnosis. The findings suggest that death from unintentional injury among patients with cancer requires further attention and that initiatives to identify patients at risk and to develop targeted prevention strategies should be prioritized.

## Key Points

**Question:**

Is the incidence of death from unintentional injury among patients with cancer higher than that in the general population?

**Findings:**

In this cohort study using Surveillance, Epidemiology, and End Results program data from 8 271 020 patients, the incidence of death from unintentional injury among patients with cancer in the United States was 60% higher than that in the general US population. The highest rates of death from unintentional injury were observed among patients with liver cancer and were within the first month after diagnosis.

**Meaning:**

The findings suggest that, for clinicians at all levels of health care, death from unintentional injury among patients with cancer requires more attention.

## Teaser

This cohort study uses Surveillance, Epidemiology, and End Results (SEER) data to evaluate the incidence of death from unintentional injury among patients with cancer compared with the general population in the United States.

## Introduction

Injury is an important cause of death among patients with cancer.^1^ An increased risk of self-inflicted injury, mainly suicide, has been observed among patients with cancer compared with the general population.^2,3^ Unintentional injuries, another major category of injury, are the third-leading causes of death in the United States.^4^ There is evidence^5,6^ to suggest that suicide and death from unintentional injury share similar risk factors, such as mental distress, physical illnesses, and impaired social and physical functioning caused by treatment. Thus, we believe that it is necessary to examine not only suicide but also death due to unintentional injury among patients with cancer. Few studies^7,8,9^ have identified increased risk of death from unintentional injury among patients with cancer. Camidge et al^7^ found a relative risk of 1.58 for death from unintentional injury among patients with cancer compared with the general Scottish population. In Japan, Yamauchi et al^8^ reported a relative risk for externally caused injury of 18.8 within the first year after cancer diagnosis. However, no large studies, to our knowledge, have examined the rates of death due to unintentional injury among patients with cancer, and it remains unclear which patient and disease characteristics, such as anatomic site of cancer, are associated with higher rates of death from unintentional injury.

The purpose of our study was to present a comprehensive analysis of death from unintentional injury among patients with cancer using a large population-based cohort. We aimed to assess the incidence of death from unintentional injury among patients with cancer and to identify subgroups of patients with cancer associated with higher rates of death from unintentional injury.

## Methods

### Data Sources

This retrospective cohort study included patients diagnosed with a first primary cancer between January 1, 1973, and December 31, 2015, identified from the Surveillance, Epidemiology, and End Results program (SEER) database.^10^ The SEER database provides information on population-based incident tumor statistics from approximately 28% of the US population who reside in various geographic locations of the United States. We used data available for public use from the SEER 18 registries.^11^ To assess comparisons with the general US population, mortality data available from the National Center for Health Statistics^11^ from January 1, 1969, to December 31, 2015, were also collected from the SEER program. The SEER data contain deidentified information and are freely available under a data use agreement with the National Cancer Institute. Thus, this study was considered to be exempt by the institutional review board of Tongji Medical College, Huazhong University of Science and Technology, Wuhan, China, and informed consent was waived. This study followed the Strengthening the Reporting of Observational Studies in Epidemiology (STROBE) reporting guideline. Analyses were performed from February 1, 2019, to August 15, 2019.

### Study Population and Study Variables

Inclusion and exclusion criteria leading to the final cohort of patients with cancer are depicted in the Figure. Patients with diagnoses solely from death certificates or autopsy were excluded. Patients were also excluded if their ages at the times of diagnosis and follow-up were unknown. Patients were observed from the time of cancer diagnosis until death due to unintentional injury, death due to other causes, or the end of this study in December 31, 2015, for which we used the SEER vital status variable and the cause-specific death classification variable.

**Figure.**
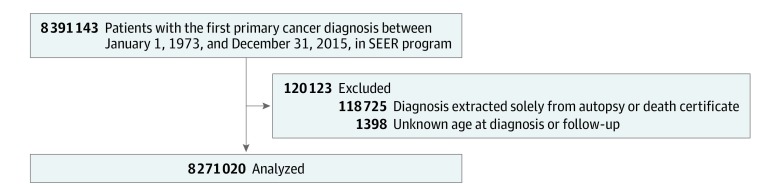
Flow Diagram of Patient Selection Within the Surveillance, Epidemiology, and End Results (SEER) Database Between 1973 and 2015

The following variables were examined: age at the time of cancer diagnosis (0-14 years, 15-19 years, 20-39 years, 40-59 years, 60-79 years, and ≥80 years), sex, race/ethnicity (white, black, American Indian or Alaska Native, Asian or Pacific Islander, and unknown), Hispanic origin (Hispanic and non-Hispanic), year of diagnosis (1973-1979, 1980-1989, 1990-1999, 2000-2009, and 2010-2015), marital status at the time of cancer diagnosis (married, unmarried, and unknown), cancer stage (in situ, localized, regional, distant, and unstaged), surgery (yes, no, and unknown), cancer site, and survival time. For stage, we used the SEER summary stage, which defines stage as localized (invasive but confined to the organ of origin), regional (extension beyond the organ of origin but no distant metastasis), or distant (distant metastasis). We did not include radiotherapy and chemotherapy as treatment variables in our analysis because data in the SEER database on radiotherapy and chemotherapy were not sensitive enough and not recommended for use in group comparisons.^12^ For patients who did not survive a full month and whose survival time was recoded as 0 months, the survival time was converted to a one-half month according to standard epidemiologic convention.^13^

### Outcome of Interest

The main outcome of interest was death from unintentional injury among patients with cancer, identified from the following cause of death codes in the SEER database: *International Classification of Diseases, Eighth Revision* codes 800-949; *International Classification of Diseases, Ninth Revision* codes 800-949; and *International Statistical Classification of Diseases and Related Health Problems, Tenth Revision* (*ICD-10*) codes V01-X59 and Y85-Y86 and recode 50210.

### Statistical Analysis

The rates of death from unintentional injury were calculated as the number of deaths from unintentional injury divided by person-years at risk. For site-specific analysis, the mortality rates of each type of cancer were adjusted to the age, sex, race/ethnicity, and calendar year distributions of all patients with a primary cancer. The entire US population with cancer was chosen as the reference category in the adjustment. For a certain type of cancer, the adjusted mortality rate was computed as a weighted average of the stratum-specific mortality rates. The weights for adjustment were the distribution of person-years over the strata of age, sex, race/ethnicity, and calendar year in all patients with a primary cancer. The age and calendar year at diagnosis were used in the adjustment. Given that they were continuous variables, age and calendar year were both divided into 5-year categories. Standardized mortality ratios (SMRs) and 95% CIs were calculated as previously described.^2,13,14^ In brief, SMRs were estimated as the ratios of observed to expected number of deaths. The observed number of deaths represents the total number of deaths from unintentional injury among patients with cancer recorded during the study period; the expected number of deaths represents the number of individuals who died from unintentional injury in the general population with a similar distribution of age at diagnosis, sex, race/ethnicity, and calendar year. To obtain the expected number of deaths, we derived the stratum-specific mortality rates from unintentional injury of the reference general population collected by the SEER program and calculated the person-years of relevant strata in the cancer group. The stratum-specific expected number of deaths was estimated as the product of mortality rate in the reference group and the person-years in the cancer group. The total expected number of deaths was a summation of all the expected number of deaths across the strata. To identify subgroups associated with a higher incidence of death from unintentional injury, we calculated the rate ratios (RRs) of different groups, which were established as a ratio of the mortality rate of the target group to the reference group. The 95% CI for SMRs and RRs were obtained based on a Poisson regression model.

All statistical tests were 2-sided, and *P* < .05 was considered to be statistically significant. Analyses were conducted with SEER*Stat software, version 8.3.5 (US Department of Health and Human Services) and R, version 3.51 (The R Project for Statistical Computing) statistical software.^10^

## Results

A total of 8 271 020 patients with cancer (50.2% female; mean [SD] age, 63.0 [15.7] years) observed for 49 571 891 person-years were included, of whom 4 502 302 died after cancer diagnosis. The median follow-up time was 3.6 years (range, 0-42.9 years). The detailed characteristics of the study participants are shown in Table 1. Unintentional injury was the cause of death in 40 599 of these patients. Among all patients with a first primary cancer, the rate of death from unintentional injury was 81.90 per 100 000 person-years. In contrast, the corresponding rate of death from unintentional injury in the US general population was 51.21 per 100 000 person-years (SMR, 1.60; 95% CI, 1.58-1.61).

**Table 1.** Death From Unintentional Injury Among Patients With Cancer by Demographic and Tumor Characteristics

Characteristic	Patients With Cancer, No. (%)	Person-Years of Follow-up	Death From Unintentional Injury				SMR (95% CI)^b^	RR (95% CI)^c^	*P* Value
			Patients With Cancer		General Population				
			Observed Deaths, No. (%)^a^	Mortality Rate, per 100 000 Person-Years	Expected Deaths, No. (%)^a^	Mortality Rate, per 100 000 Person-Years			
Overall	8 271 020 (100)	49 571 891	40 599 (100)	81.90	25 385.6	51.21	1.60 (1.58-1.61)	NA	NA
Age at diagnosis, y									
0-14	70 918 (0.9)	670 265	172 (0.4)	25.66	87.0	12.98	1.98 (1.70-2.30)	0.26 (0.23-0.31)	<.001
15-19	35 104 (0.4)	350 832	143 (0.4)	40.76	133.7	38.11	1.07 (0.91-1.26)	0.42 (0.36-0.49)	<.001
20-39	509 320 (6.2)	4 965 279	1836 (4.5)	36.98	1535.3	30.92	1.20 (1.14-1.25)	0.38 (0.36-0.40)	<.001
40-59	2 468 035 (29.8)	18 374 139	8182 (20.2)	44.53	5976.5	32.53	1.37 (1.34-1.40)	0.46 (0.45-0.47)	<.001
60-79	4 055 038 (49.0)	22 101 157	21 475 (52.9)	97.17	11 468.2	51.89	1.87 (1.85-1.90)	1 [Reference]	NA
≥80	1 132 605 (13.7)	3 110 219	8791 (21.7)	282.65	6184.9	198.86	1.42 (1.39-1.45)	2.91 (2.84-2.98)	<.001
Sex									
Female	4 154 399 (50.2)	27 009 545	16 818 (41.4)	62.27	9276.4	34.34	1.81 (1.79-1.84)	1 [Reference]	NA
Male	4 116 621 (49.8)	22 562 346	23 781 (58.6)	105.40	16 109.3	71.40	1.48 (1.46-1.50)	1.69 (1.66-1.73)	<.001
Race/ethnicity^d^									
White	5 740 618 (69.4)	32 171 107	26 929 (66.3)	83.71	17 131.9	53.25	1.57 (1.55-1.59)	1 [Reference]	NA
Black	723 872 (8.8)	3 462 970	2540 (6.3)	73.35	1893.6	54.68	1.34 (1.29-1.39)	0.88 (0.84-0.91)	<.001
American Indian or Alaska Native	37 945 (0.5)	188 551	233 (0.6)	123.57	116.9	62.00	1.99 (1.75-2.27)	1.48 (1.30-1.68)	<.001
Asian or Pacific Islander	452 926 (5.5)	2 405 246	1310 (3.2)	54.46	677.1	28.15	1.93 (1.83-2.04)	0.65 (0.62-0.69)	<.001
Unknown	96 963 (1.2)	486 437	97 (0.2)	19.94	NA	NA	NA	NA	NA
Hispanic origin									
Non-Hispanic	7 584 447 (91.7)	46 005 352	38 450 (94.7)	83.58	23 758.5	51.64	1.62 (1.60-1.63)	1 [Reference]	NA
Hispanic	686 573 (8.3)	3 566 539	2149 (5.3)	60.25	1627.2	45.62	1.32 (1.27-1.38)	0.72 (0.69-0.75)	<.001
Year of diagnosis									
1973-1979	427 455 (5.2)	3 809 616	3180 (7.8)	83.47	1995.5	52.38	1.59 (1.54-1.65)	1.09 (1.05-1.13)	<.001
1980-1989	791 241 (9.6)	7 047 964	6310 (15.5)	89.53	3304.0	46.88	1.91 (1.86-1.96)	1.17 (1.13-1.20)	<.001
1990-1999	1 327 978 (16.1)	11 931 280	9781 (24.1)	81.98	5512.0	46.20	1.77 (1.74-1.81)	1.07 (1.04-1.09)	<.001
2000-2009	3 444 331 (41.6)	21 897 895	16 816 (41.4)	76.79	11 523.1	52.62	1.46 (1.44-1.48)	1 [Reference]	NA
2010-2015	2 280 015 (27.6)	4 885 135	4512 (11.1)	92.36	3051.0	62.46	1.48 (1.44-1.52)	1.20 (1.16-1.24)	<.001
Marital status									
Married	4 563 510 (55.2)	30 318 658	21 370 (52.6)	70.48	14 863.1	49.02	1.44 (1.42-1.46)	1 [Reference]	NA
Unmarried	3 089 379 (37.4)	3 625 370	16 088 (39.6)	86.64	8464.4	56.77	1.90 (1.87-1.93)	1.23 (1.18-1.28)	<.001
Unknown	618 131 (7.5)	15 627 863	3141 (7.7)	102.94	2058.1	54.16	1.53 (1.47-1.58)	1.46 (1.43-1.49)	<.001
Cancer stage									
In situ	505 512 (6.1)	4 375 845	2462 (6.1)	56.26	1746.2	39.90	1.41 (1.36-1.47)	0.78 (0.75-0.82)	<.001
Localized	3 210 553 (38.8)	26 054 162	18 728 (46.1)	71.88	13 325.8	51.15	1.41 (1.39-1.43)	1 [Reference]	NA
Regional	1 412 579 (17.1)	7 997 811	6431 (15.8)	80.41	3653.6	45.68	1.76 (1.72-1.80)	1.12 (1.09-1.15)	<.001
Distant	1 481 379 (17.9)	3 156 920	3745 (9.2)	118.63	1658.5	52.54	2.26 (2.19-2.33)	1.65 (1.59-1.71)	<.001
Unstaged	1 660 997 (20.1)	7 987 153	9233 (22.7)	115.60	5001.5	62.62	1.85 (1.81-1.88)	1.61 (1.57-1.65)	<.001
Surgery									
Yes	4 947 428 (59.8)	38 199 670	26 245 (64.6)	68.70	17 777.1	46.54	1.48 (1.46-1.49)	1 [Reference]	NA
No	3 187 762 (38.5)	10 868 054	13 765 (33.9)	126.66	7306.1	67.23	1.88 (1.85-1.92)	1.84 (1.81-1.88)	<.001
Unknown	135 830 (1.6)	504 167	589 (1.5)	116.83	302.4	59.98	1.95 (1.80-2.11)	1.70 (1.57-1.85)	<.001

Abbreviations: NA, not applicable; RR, rate ratio; SMR, standardized mortality ratio.

^a^ Observed deaths represent the total number of deaths from unintentional injury among patients with cancer recorded during the study period. Expected deaths represent the number of individuals who died of unintentional injury in the general population with a similar distribution of age at diagnosis (5-year intervals), sex, race/ethnicity (white, black, and other), and calendar year of diagnosis (5-year intervals).

^b^ Estimated as the ratios of observed to expected number of deaths. For the categories of age, sex, and year of diagnosis, the SMR reference population was the specific category in the US subpopulation from 1973 through 2015. For race/ethnicity, the specific race/ethnicity codes (white, black, American Indian or Alaska Native, and Asian or Pacific Islander) were used.

^c^ Rate ratios were calculated as the ratios of the crude mortality rate in a certain group to that in the reference group.

^d^ For race, patients diagnosed before 1990 were excluded because the Surveillance, Epidemiology, and End Results program began to report data regarding ethnicity in 1990.

### Patient Characteristics

Higher rates of death from unintentional injury among patients with cancer were associated with increasing age at diagnosis (>80 years; RR, 2.91; 95% CI, 2.84-2.98; *P* < .001), male sex (RR, 1.69; 95% CI, 1.66-1.73; *P* < .001), American Indian or Alaska Native population (RR, 1.48; 95% CI, 1.30-1.68; *P* < .001), non-Hispanic origin (RR, 1.39; 95% CI, 1.33-1.45; *P* < .001), and being unmarried (RR, 1.23; 95% CI, 1.18-1.28; *P* < .001) (Table 1). Although only 3745 patients (9.2%) had advanced disease, the highest rate of death from unintentional injury was among these patients (118.63 per 100 000 person-years). Rates of death from unintentional injury were higher among patients diagnosed before 1990, decreased gradually thereafter, and increased again among patients diagnosed from 2010 to 2015.

### Tumor Sites

For 28 of 29 cancer sites (all except the testis), rates of death from unintentional injury were higher among patients with cancer than in the general population (Table 2). After adjustment for age at diagnosis, race/ethnicity, sex, and calendar year, rates of death from unintentional injury were the highest among patients with liver cancer (200.37 per 100 000 person-years; SMR, 4.09; 95% CI, 3.68-4.54), followed by brain cancer (175.04 per 100 000 person-years; SMR, 2.41; 95% CI, 2.18-2.68), laryngeal cancer (148.78 per 100 000 person-years; SMR, 2.27; 95% CI, 2.10-2.46), and esophagus cancer (144.98 per 100 000 person-years; SMR, 2.52; 95% CI, 2.20-2.88). Compared with the general population, the highest risk of death from unintentional injury was observed among patients with liver cancer (RR, 2.10; 95% CI, 1.86-2.37; *P* < .001).

**Table 2.** Death From Unintentional Injury by Anatomic Site of Cancer^a^

Anatomic Site	Patients With Cancer, No.	Survival Time, Person-Years	Observed Deaths in Patients With Cancer, No. (%)^b^	Mortality Rate, per 100 000 Person-Years^c^	Expected Deaths in General Population^d^^,^^e^	SMR (95% CI)^e^^,^^f^	Rate Ratio (95% CI)^g^	*P* Value
Liver	110 062	151 540	347	200.37	84.8	4.09 (3.68-4.54)	2.10 (1.86-2.37)	<.001
Brain	119 951	433 152	360	175.04	149.1	2.41 (2.18-2.68)	1.87 (1.72-2.03)	<.001
Larynx	68 477	448 253	626	148.78	275.5	2.27 (2.10-2.46)	1.56 (1.43-1.71)	<.001
Esophagus	71 098	128 516	216	144.98	85.8	2.52 (2.20-2.88)	1.52 (1.31-1.77)	<.001
Myeloma	97 618	328 220	464	130.25	202.4	2.29 (2.09-2.51)	1.37 (1.23-1.52)	<.001
Lung and bronchus	941 791	1 736 764	2516	128.60	1023.9	2.46 (2.36-2.56)	1.35 (1.27-1.43)	<.001
Leukemia	214 713	1 027 293	1132	123.22	521.3	2.17 (2.05-2.30)	1.31 (1.22-1.41)	<.001
Oral cavity and pharynx	188 740	1 078 200	1363	121.79	596.5	2.28 (2.17-2.41)	1.28 (1.19-1.37)	<.001
Pancreas	188 750	170 723	247	121.64	109.3	2.26 (2.00-2.56)	1.28 (1.11-1.47)	<.001
Small intestine	27 886	134 104	140	115.33	72.1	1.94 (1.65-2.29)	1.21 (1.03-1.43)	.01
Stomach	136 907	348 706	441	112.60	230.9	1.91 (1.74-2.10)	1.18 (1.06-1.32)	.001
Anus, anal canal, and anorectum	33 161	193 730	176	104.43	91.9	1.92 (1.65-2.22)	1.10 (0.95-1.27)	.11
Kidney and renal pelvis	205 813	1 127 572	1071	94.52	621.0	1.72 (1.62-1.83)	0.99 (0.92-1.07)	.42
Lymphoma	355 580	2 222 105	1812	93.84	1107.3	1.64 (1.56-1.71)	1 [Reference]	NA
Eye and orbit	14 345	110 083	90	92.11	61.3	1.47 (1.19-1.80)	0.97 (0.79-1.18)	.37
Bones and joints	16 369	125 761	84	89.41	46.0	1.83 (1.47-2.26)	0.95 (0.79-1.15)	.31
Breast	1 343 540	11 458 385	5968	86.12	3746.4	1.59 (1.55-1.63)	0.90 (0.86-0.95)	<.001
Other nervous system including cranial nerves	110 098	505 274	495	80.66	276.9	1.79 (1.64-1.95)	0.85 (0.76-0.94)	.001
Soft tissue including heart	50 345	339 247	215	80.40	156.88	137 (1.20-1.57)	0.86 (0.76-0.97)	.01
Urinary bladder	304 521	2 053 140	2248	80.20	1515.6	1.48 (1.42-1.55)	0.84 (0.79-0.90)	<.001
Colon and rectum	824 153	4 936 424	4911	79.66	3120.5	1.57 (1.53-1.62)	0.84 (0.79-0.88)	<.001
Endocrine system	220 146	1 729 526	651	78.26	559.6	1.16 (1.08-1.26)	0.82 (0.77-0.88)	<.001
Skin, non-basal	491 679	3 962 943	2242	70.37	1986.5	1.13 (1.08-1.18)	0.74 (0.70-0.78)	<.001
Vulva	48 147	421 438	309	44.68	126.9	2.43 (2.18-2.72)	0.47 (0.40-0.54)	<.001
Prostate	1 137 848	8 625 118	8365	39.68	6424.8	1.30 (1.27-1.33)	0.42 (0.39-0.44)	<.001
Cervix uteri	87 245	771 211	331	37.66	171.8	1.93 (1.73-2.15)	0.40 (0.35-0.45)	<.001
Testis	52 616	606 739	315	36.51	344.2	0.92 (0.82-1.02)	0.39 (0.34-0.45)	<.001
Ovary	128 056	712 331	322	29.58	198.3	1.62 (1.46-1.81)	0.31 (0.27-0.36)	<.001
Corpus and uterus, not otherwise specified	245 483	2 183 171	1195	29.00	673.4	1.77 (1.68-1.88)	0.30 (0.28-0.33)	<.001

Abbreviation: SMR, standardized mortality ratio.

^a^ Analysis was limited to tumor sites at least 100 000 person-years. For adult-onset cancers, mortality rates and SMRs were calculated in patients with cancer aged 20 years or older; for cancers of brain, leukemias, lymphomas, cancers of bone and joint, cancers of soft tissue, and cancers of testis, mortality rates were calculated in all ages.

^b^ The total number of deaths from unintentional injury in patients with cancer recorded during the study period.

^c^ Adjusted to the age at diagnosis, race, sex, and calendar year of diagnosis distributions of all patients with the first primary tumor.

^d^ The number of individuals who died of unintentional injury in the general population with a similar distribution of age at diagnosis (5-year intervals), sex, race/ethnicity (white, black, and other), and calendar year of diagnosis (5-year age intervals).

^e^ Reference population is defined as the general US population, 1973 to 2015.

^f^ Estimated as the ratios of observed to expected number of deaths.

^g^ Calculated as the ratios of the mortality rates from unintentional injury in patients with a certain cancer to that in patients with lymphoma.

### Risk of Death

For most cancer types, the SMRs of death from unintentional injury were the highest in the first month after cancer diagnosis, decreased gradually from 1 month to 5 years, and increased after 5 years (Table 3). For certain cancers (eg, breast, prostate, skin, urinary bladder, vulva, and corpus uteri), the SMRs of death from unintentional injury were higher during the 5 years after diagnosis than within the first month.

**Table 3.** Death From Unintentional Injury Among Patients With Cancer by Site and Time Since Diagnosis^a^

Cancer Site, Time Since Diagnosis	Person-Years Accrued^b^	Deaths From Unintentional Injury, No./Total No. (%)	SMR (95% CI)^c^
**All**			
Within 1 mo	669 147	1489/40 599 (3.7)	3.21 (3.05-3.38)
2-12 mo	5 607 093	5218/40 599 (12.9)	1.45 (1.41-1.49)
13 mo to 5 y	18 349 230	12 421/40 599 (30.6)	1.18 (1.16-1.20)
>5 y	24 959 086	21 471/40 599 (52.9)	1.99 (1.97-2.02)
**Liver**			
Within 1 mo	8314	54/347 (15.6)	9.18 (7.03-11.99)
2-12 mo	44 702	121/347 (34.9)	4.26 (3.56-5.09)
13 mo to 5 y	68 233	138/347 (39.8)	3.66 (3.10-4.32)
>5 y	30 812	34/347 (9.8)	2.57 (1.83-3.59)
**Brain**			
Within 1 mo	9602	22/360 (6.1)	4.29 (2.82-6.51)
2-12 mo	68 347	73/360 (20.3)	2.48 (1.97-3.12)
13 mo to 5 y	157 163	119/360 (33.1)	2.16 (1.81-2.59)
>5 y	198 350	146/360 (40.6)	2.44 (2.08-2.87)
**Larynx**			
Within 1 mo	5646	7/626 (1.1)	1.71 (0.82-3.60)
2-12 mo	50 870	85/626 (13.6)	2.38 (1.93-2.95)
13 mo to 5 y	166 274	207/626 (33.1)	1.88 (1.64-2.16)
>5 y	225 513	327/626 (52.2)	2.60 (2.33-2.89)
**Esophagus**			
Within 1 mo	5663	19/216 (8.8)	4.02 (2.57-6.31)
2-12 mo	35 730	68/216 (31.5)	2.49 (1.96-3.16)
13 mo to 5 y	52 573	71/216 (32.9)	2.01 (1.59-2.54)
>5 y	34 779	58/216 (26.9)	3.11 (2.40-4.02)
**Myeloma**			
Within 1 mo	7874	40/464 (8.6)	6.38 (4.68-8.69)
2-12 mo	63 854	95/464 (20.5)	2.04 (1.67-2.49)
13 mo to 5 y	166 638	209/464 (45.0)	1.98 (1.73-2.26)
>5 y	90 031	120/464 (25.9)	2.72 (2.28-3.26)
**Lung and Bronchus**			
Within 1 mo	73 393	291/2516 (11.6)	5.18 (4.62-5.81)
2-12 mo	444 261	768/2516 (30.5)	2.51 (2.34-2.69)
13 mo to 5 y	705 835	790/2516 (31.4)	1.86 (1.73-1.99)
>5 y	516 886	667/2516 (26.5)	2.78 (2.58-3.00)
**Leukemia**			
Within 1 mo	16 894	109/1132 (9.6)	8.54 (7.08-10.30)
2-12 mo	132 969	247/1132 (21.8)	2.78 (2.46-3.15)
13 mo to 5 y	402 700	418/1132 (36.9)	1.80 (1.63-1.98)
>5 y	475 156	358/1132 (31.6)	1.91 (1.72-2.11)
**Oral Cavity and Pharynx**			
Within 1 mo	15 538	22/1363 (1.6)	2.03 (1.34-3.09)
2-12 mo	135 986	204/1363 (15.0)	2.26 (1.97-2.59)
13 mo to 5 y	409 425	492/1363 (36.1)	2.00 (1.83-2.18)
>5 y	517 422	645/1363 (47.3)	2.59 (2.40-2.80)
**Pancreas**			
Within 1 mo	14 241	33/247 (13.4)	2.72 (1.93-3.82)
2-12 mo	65 797	115/247 (46.6)	2.45 (2.04-2.94)
13 mo to 5 y	60 994	61/247 (24.7)	1.66 (1.29-2.13)
>5 y	30 738	38/247 (15.4)	2.64 (1.92-3.63)
**Small Intestine**			
Within 1 mo	2245	12/140 (8.6)	7.69 (4.36-13.53)
2-12 mo	18 529	25/140 (17.9)	2.12 (1.43-3.13)
13 mo to 5 y	56 640	37/140 (26.4)	1.13 (0.82-1.56)
>5 y	56 737	66/140 (47.1)	2.53 (1.99-3.22)
**Stomach**			
Within 1 mo	10 778	28/441 (6.3)	2.96 (2.04-4.29)
2-12 mo	69 902	111/441 (25.2)	2.02 (1.68-2.43)
13 mo to 5 y	136 959	135/441 (30.6)	1.41 (1.19-1.67)
>5 y	131 547	167/441 (37.9)	2.34 (2.01-2.72)
**Anus, Anal Canal, and Anorectum**			
Within 1 mo	2735	3/176 (1.7)	1.89 (0.61-5.85)
2-12 mo	24 695	28/176 (15.9)	2.05 (1.41-2.96)
13 mo to 5 y	80 373	56/176 (31.8)	1.37 (1.06-1.78)
>5 y	85 949	89/176 (50.6)	2.48 (2.02-3.06)
**Lymphoma**			
Within 1 mo	28 771	76/1812 (4.2)	3.84 (3.07-4.81)
2-12 mo	243 083	320/1812 (17.7)	2.10 (1.88-2.34)
13 mo to 5 y	813 535	533/1812 (29.4)	1.19 (1.09-1.29)
>5 y	1 137 212	883/1812 (48.7)	1.81 (1.70-1.94)
**Kidney and Renal Pelvis**			
Within 1 mo	16 756	54/1071 (5.0)	4.70 (3.60-6.14)
2-12 mo	141 313	152/1071 (14.2)	1.67 (1.42-1.95)
13 mo to 5 y	457 695	336/1071 (31.4)	1.24 (1.11-1.38)
>5 y	512 104	529/1071 (49.4)	2.14 (1.96-2.33)
**Eye and Orbit**			
Within 1 mo	1185	0/90	NA
2-12 mo	11 165	6/90 (6.7)	0.77 (0.35-1.72)
13 mo to 5 y	40 228	40/90 (44.4)	1.57 (1.15-2.14)
>5 y	57 510	44/90 (48.9)	1.61 (1.20-2.17)
**Bones and Joints**			
Within 1 mo	1348	1/84 (1.2)	1.65 (0.23-11.71)
2-12 mo	12 142	11/84 (13.1)	2.19 (1.22-3.96)
13 mo to 5 y	39 674	25/84 (29.8)	1.68 (1.14-2.49)
>5 y	72 609	47/84 (56.0)	1.84 (1.38-2.45)
**Breast**			
Within 1 mo	111 241	62/5968 (1.0)	1.32 (1.03-1.69)
2-12 mo	1 055 680	382/5968 (6.4)	0.88 (0.79-0.97)
13 mo to 5 y	4 024 018	1621/5968 (27.2)	1.06 (1.01-1.12)
>5 y	6 267 956	3903/5968 (65.4)	2.24 (2.17-2.31)
**Soft Tissue Including Heart**			
Within 1 mo	3634	6/215 (2.8)	2.43 (1.09-5.41)
2-12 mo	35 833	23/215 (10.7)	1.10 (0.73-1.66)
13 mo to 5 y	114 900	64/215 (29.8)	1.08 (0.84-1.38)
>5 y	184 450	122/215 (56.7)	1.65 (1.38-1.96)
**Cranial Nerves and Other Nervous System**			
Within 1 mo	8992	79/495 (16.0)	12.73 (10.21-15.88)
2-12 mo	80 202	103/495 (20.8)	1.97 (1.63-2.40)
13 mo to 5 y	263 499	190/495 (38.4)	1.26 (1.09-1.45)
>5 y	152 676	123/495 (24.8)	1.82 (1.53-2.18)
**Urinary Bladder**			
Within 1 mo	25 049	38/2248 (1.7)	1.50 (1.10-2.07)
2-12 mo	224 684	217/2248 (9.7)	1.01 (0.88-1.15)
13 mo to 5 y	768 511	699/2248 (31.1)	1.07 (1.00-1.16)
>5 y	1 035 143	1294/2248 (57.6)	2.07 (1.96-2.19)
**Colon and Rectum**			
Within 1 mo	66 791	145/4911 (3.0)	2.60 (2.21-3.06)
2-12 mo	572 266	546/4911 (11.1)	1.23 (1.13-1.33)
13 mo to 5 y	1 850 609	1407/4911 (28.6)	1.07 (1.02-1.13)
>5 y	2 447 885	2813/4911 (57.3)	2.15 (2.07-2.23)
**Endocrine System**			
Within 1 mo	18 139	19/651 (2.9)	2.69 (1.71-4.21)
2-12 mo	168 187	66/651 (10.1)	1.04 (0.82-1.32)
13 mo to 5 y	626 797	203/651 (31.2)	0.91 (0.80-1.05)
>5 y	916 527	363/651 (55.8)	1.36 (1.23-1.51)
**Skin Excluding Basal and Squamous Cell**			
Within 1 mo	40 323	23/2242 (1.0)	0.86 (0.57-1.29)
2-12 mo	375 788	186/2242 (8.3)	0.76 (0.66-0.88)
13 mo to 5 y	1 404 571	729/2242 (32.5)	0.89 (0.83-0.96)
>5 y	2 142 504	1304/2242 (58.2)	1.45 (1.37-1.53)
**Vulva**			
Within 1 mo	3972	0/309	NA
2-12 mo	37 053	22/309 (7.1)	1.39 (0.91-2.11)
13 mo to 5 y	141 123	88/309 (28.5)	1.74 (1.42-2.15)
>5 y	239 313	199/309 (64.4)	3.38 (2.94-3.89)
**Prostate**			
Within 1 mo	94 085	103/8365 (1.2)	1.25 (1.03-1.52)
2-12 mo	895 132	590/8365 (7.1)	0.77 (0.71-0.84)
13 mo to 5 y	3 459 819	2632/8365 (31.5)	0.95 (0.92-0.99)
>5 y	4 176 472	5040/8365 (60.3)	1.79 (1.74-1.84)
**Cervix Uteri**			
Within 1 mo	7188	5/331 (1.5)	2.29 (0.95-5.50)
2-12 mo	64 728	38/331 (11.5)	2.07 (1.51-2.85)
13 mo to 5 y	220 423	75/331 (22.7)	1.36 (1.09-1.71)
>5 y	478 937	213/331 (64.4)	2.21 (1.93-2.53)
**Testis**			
Within 1 mo	4346	0/315	NA
2-12 mo	41 231	47/315 (14.9)	2.03 (1.53-2.71)
13 mo to 5 y	166 366	80/315 (25.4)	0.86 (0.69-1.08)
>5 y	394 814	188/315 (59.7)	0.83 (0.72-0.96)
**Ovary**			
Within 1 mo	10 270	9/322 (2.8)	1.89 (0.99-3.64)
2-12 mo	83 955	53/322 (16.5)	1.66 (1.27-2.17)
13 mo to 5 y	245 101	94/322 (29.2)	1.22 (0.99-1.49)
>5 y	373 244	166/322 (51.6)	1.96 (1.69-2.29)
**Corpus and Uterus, Not Otherwise Specified**			
Within 1 mo	20 250	19/1195 (1.6)	2.29 (1.46-3.59)
2-12 mo	185 812	75/1195 (6.3)	1.03 (0.82-1.29)
13 mo to 5 y	677 783	211/1195 (17.7)	0.88 (0.77-1.01)
>5 y	1 299 498	890/1195 (74.5)	2.52 (2.36-2.69)

Abbreviations: NA, not applicable; SMR, standardized mortality ratio.

^a^ Analysis was limited to tumor sites for which at least 100 000 person-years were accrued.

^b^ For a certain time interval after diagnosis, the follow-up time of patients was calculated as the beginning of the interval to death from unintentional injury, death from other causes, or exit from the study or the end of the interval; the accrued person-years was then a summation of the follow-up time of all patients. Calculating the person-years of the 1-to 5-year interval, and the accrued person-years were then a summation of the follow-up time of all patients.

^c^ Reference population was defined as the general US population, 1973 to 2015. The SMRs were estimated as the ratios of observed to expected number of deaths. The observed deaths represent the total number of deaths from unintentional injury among patients with cancer recorded during the study period. The expected number of deaths represents the number of individuals who died of unintentional injury in the general population with a similar distribution of age at diagnosis (5-year intervals), sex, race/ethnicity (white, black, and other), and calendar year of diagnosis (5-year age intervals).

## Discussion

In this study, we comprehensively analyzed death from unintentional injury among more than 8.2 million patients with cancer using nationally representative data from the SEER program. We found that the incidence of death from unintentional injury among patients from the United States who had cancer was approximately 60% higher than that in the general US population, and we identified types of cancer and groups of patients with a higher incidence of death from unintentional injury.

Patients diagnosed with cancer often experience psychologic stress,^15,16^ physical illnesses or disabilities,^17^ social dysfunction,^18^ substance abuse,^19^ and poor quality of life,^20^ all of which may be associated with an increased risk of death from unintentional injury. The results of the present study showed that patients with nearly all types of cancers were at an increased risk of death from unintentional injury. Of note, we found that patients with cancers of the liver, larynx, and esophagus had the highest rates of death from unintentional injury. These cancers are associated with alcohol consumption.^21^ Patients with cancers associated with alcohol consumption may be at a higher risk of unintentional injuries.^22,23^ The reasons for the association of these cancers with increased rate of death from unintentional injury warrant further investigation. In addition, brain cancer was identified as another type of cancer that was associated with a high rate of death from unintentional injury. Possible hypotheses to explain this association include physical disability,^24^ cognitive dysfunction,^25^ mental distress,^26^ and epilepsy^27^ in patients with brain cancer.

We observed that the risk of death from unintentional injury in patients with cancer was the highest immediately after diagnosis. Similarly, Yamauchi et al^8^ reported a relative risk of 18.8 within the first year after cancer diagnosis and a sharply decreased relative risk of 1.2 beyond the first year after diagnosis in a population-based study in Japan. These findings highlight the concept that the diagnosis of cancer might be a major stressor that immediately affects the risk of fatal outcomes.^28^

Older age was associated with higher rate of death from unintentional injury in our analysis. Older patients are more likely to have inattention, slow reaction, impaired vision or hearing, advanced disease, and other medical complications, all of which are associated with an increased risk of unintentional injuries, including falls and traffic accidents. Our study also showed that race/ethnicity may be significantly associated with the risk of death from unintentional injury. Patients of the American Indian or Alaskan Native population had the highest rates of death from unintentional injury compared with other races/ethnicities. This racial difference, which is consistent with that noted in the general American population by the National Center for Health Statistics,^29^ may be associated with multiple risk factors, including behaviors, policies, and certain socioeconomic factors. Male sex and advanced stage of cancer were associated with a higher incidence of death from unintentional injury, which was similar to the results of a previous study.^30^ In addition, our study showed that patients with cancer who were unmarried were more likely to have increased mortality due to unintentional injury. Furthermore, emotional support from a partner has been associated with decreased risk of death from unintentional injury.^31^

Most patients with cancer now die of noncancer causes.^32^ Our data suggest that death due to unintentional injury in cancer deserves further attention. Although some unintentional injuries are not completely preventable, our results showed that patients with cancer are at an increased risk of death due to unintentional injury, providing evidence for physicians, allied health professionals, and others to formulate plans for ways to prevent death from unintentional injury in this population. Further research is warranted to identify specific risk factors for the different categories of death from unintentional injury.

### Limitations

This study has limitations. First, deaths from unintentional injury constitute a group of fatal events that are qualitatively different (eTable in the Supplement). For instance, accidental poisonings by narcotic substances are likely to be associated with illicit drug use or addiction, distinguishing this group from other causes of death from unintentional injury.^33^ However, SEER does not specify the precise cause of death from unintentional injury, and we were unable to focus on specific categories of death from unintentional injury among patients with cancer. In addition, the inclusion of sequelae of unintentional injuries (*ICD-10* codes Y85-Y86) may not be appropriate because these events can occur before cancer diagnosis. In our study, death from unintentional injury was analyzed as a whole.

Second, the risk of reporting bias in death certificates may lead to misclassification of causes of death.^34,35^ Thus, it is difficult to distinguish between death from unintentional injury due to suicide and homicide.^6,36^ For instance, poisonings are easily misclassified as unintentional injuries instead of suicides.^37,38^ Because the literature pertaining to the misclassification of the cause of death is limited, we were unable to account for this confounding factor. Nevertheless, systematic and standardized data collection procedures are used to ensure that the causes of death recoded in SEER are accurate^39^; thus, we believe that the present study presents a reliable picture of death from unintentional injury among patients with cancer in the United States.

Third, data on comorbidities, psychiatric conditions, performance status, quality of life, employment status, social support, dependence on alcohol, tobacco consumption, and other forms of substance abuse are unavailable in SEER; thus, we could not assess the possibility of an association of these factors with the risk of death from unintentional injury. Analyzing the extensive amount of available data from the SEER database remains a powerful, useful, and integral tool in medical research for the purpose of exploratory analyses.^35^

## Conclusions

These findings suggest for the first time, to our knowledge, that the incidence of death from unintentional injury among patients with cancer is significantly higher than that in the general population in the United States. Clinicians at all levels of health care should be aware of the potential for death from unintentional injury among patients with cancer and its associated risk factors. Our results suggest the need for targeted preventive interventions of unintentional injuries among patients with cancer.

## Notes

Supplement. **eTable.** Detailed *ICD* Codes of Death From Unintentional Injury (Accidents and Adverse Effects)
