# Supplementary material for: Incidence of Death From Unintentional Injury Among Patients With Cancer in the United States
Source: JAMA Netw Open. 2020 Feb 21;3(2):e1921647. doi: 10.1001/jamanetworkopen.2019.21647 (PMC7043194; doi:10.1001/jamanetworkopen.2019.21647)
Supplement: Supplement. — eTable. Detailed ICD Codes of Death From Unintentional Injury (Accidents and Adverse Effects) [file jamanetwopen-3-e1921647-s001.pdf]

Supplementary Online Content

Yang K, Zheng Y, Peng J, et al. Incidence of death from unintentional injury among patients with cancer in the United States. *JAMA Netw Open*. 2020;3(2)e1921647. doi:10.1001/jamanetworkopen.2019.21647

eTable. Detailed *ICD* Codes of Death From Unintentional Injury (Accidents and Adverse Effects)

This supplementary material has been provided by the authors to give readers additional information about their work.

© 2020 Yang K et al. *JAMA Network Open.*

eTable. Detailed ICD codes of death from unintentional injury (Accidents and Adverse Effects).

ICD-8 codes (1973-1978)	
ICD-8 codes	Detail
E800-E807	Railway accidents
E810-E819	Motor vehicle traffic accidents
E820-E823	Motor vehicle non-traffic accidents
E825-E827	Other road vehicle accidents
E830-E838	Water transport accidents
E840-E845	Air and space transport accidents
E850-E859	Accidental poisoning by drugs and medicaments
E860-E869	Accidental poisoning by other solid and liquid substances
E870-E877	Accidental poisoning by gases and vapours
E880-E887	Accidental falls
E890-E899	Accidents caused by fires and flames
E900-E909	Accidents due to natural and environmental factors
E910-E929	Other accidents
E930-E936	Surgical and medical complications and misadventures
E940-E949	Late effects of accidental injury
ICD-9 codes (1979-1998)	
ICD-9 codes	Detail
E800–E807	Railway accidents
E810–E819	Motor vehicle traffic accidents
E820–E825	Motor vehicle non-traffic accidents
E826–E829	Other road vehicle accidents
E830–E838	Water transport accidents
E840–E845	Air and space transport accidents
E846–E848	Vehicle accidents not elsewhere classifiable
E849	Place of Occurrence
E850–E858	Accidental poisoning by drugs, medicinal substances, and biologicals
E860–E869	Accidental poisoning by other solid and liquid substances, gases, and vapors
E870–E876	Misadventures to patients during surgical and medical care
E878–E879	Surgical and medical procedures as the cause of abnormal reaction of patient or later complication, without mention of misadventure at the time of procedure
E880–E888	Accidental falls
E890–E899	Accidents caused by fire and flames
E900–E909	Accidents due to natural and environmental factors
E910–E915	Accidents caused by submersion, suffocation, and foreign bodies
E916–E928	Other accidents
E929	Late effects of accidental injury
E930–E949	Drugs, medicinal and biological substances causing adverse effects in therapeutic use
ICD-10 codes (1999+)	

© 2020 Yang K et al. *JAMA Network Open.*

ICD-10 codes	Details
V01-V99	Accidents
V01-V09	Pedestrian injured in transport accident
V10-V19	Pedal cyclist injured in transport accident
V20-V29	Motorcycle rider injured in transport accident
V30-V39	Occupant of three-wheeled motor vehicle injured in transport accident
V40-V49	Car occupant injured in transport accident
V50-V59	Occupant of pick-up truck or van injured in transport accident
V60-V69	Occupant of heavy transport vehicle injured in transport accident
V70-V79	Bus occupant injured in transport accident
V80-V89	Other land transport accidents
V90-V94	Water transport accidents
V95-V97	Air and space transport accidents
V98-V99	Other and unspecified transport accidents
W00-X59	Other external causes of accidental injury
W00-W19	Falls
W20-W49	Exposure to inanimate mechanical forces
W50-W64	Exposure to animate mechanical forces
W65-W74	Accidental drowning and submersion
W75-W84	Other accidental threats to breathing
W85-W99	Exposure to electric current, radiation and extreme ambient air temperature and pressure
X00-X09	Exposure to smoke, fire and flames
X10-X19	Contact with heat and hot substances
X20-X29	Contact with venomous animals and plants
X30-X39	Exposure to forces of nature
X40-X49	Accidental poisoning by and exposure to noxious substances
X50-X57	Overexertion, travel and privation
X58-X59	Accidental exposure to other and unspecified factors
Y85-Y86	Sequelae of accidents
Y85	Sequelae of transport accidents
Y86	Sequelae of other accidents

© 2020 Yang K et al. *JAMA Network Open.*
